# Characteristics and Outcomes of Contacts of COVID-19 Patients Monitored Using an Automated Symptom Monitoring Tool — Maine, May–June 2020

**DOI:** 10.15585/mmwr.mm6931e2

**Published:** 2020-08-07

**Authors:** Anna Krueger, Jayleen K. L. Gunn, Joanna Watson, Andrew E. Smith, Rebecca Lincoln, Sara L. Huston, Emilio Dirlikov, Sara Robinson

**Affiliations:** ^1^Maine Center for Disease Control and Prevention, Augusta, Maine; ^2^CDC COVID-19 Response Team; ^3^University of Southern Maine, Portland, Maine.

SARS-CoV-2, the virus that causes coronavirus disease 2019 (COVID-19), is spread from person to person (*1*–*3*). Quarantine of exposed persons (contacts) for 14 days following their exposure reduces transmission (*4*–*7*). Contact tracing provides an opportunity to identify contacts, inform them of quarantine recommendations, and monitor their symptoms to promptly identify secondary COVID-19 cases (*7*,*8*). On March 12, 2020, Maine Center for Disease Control and Prevention (Maine CDC) identified the first case of COVID-19 in the state. Because of resource constraints, including staffing, Maine CDC could not consistently monitor contacts, and automated technological solutions for monitoring contacts were explored. On May 14, 2020, Maine CDC began enrolling contacts of patients with reported COVID-19 into Sara Alert (MITRE Corporation, 2020),* an automated, web-based, symptom monitoring tool. After initial communication with Maine CDC staff members, enrolled contacts automatically received daily symptom questionnaires via their choice of e-mailed weblink, text message, texted weblink, or telephone call until completion of their quarantine. Epidemiologic investigations were conducted for enrollees who reported symptoms or received a positive SARS-CoV-2 test result. During May 14–June 26, Maine CDC enrolled 1,622 contacts of 614 COVID-19 patients; 190 (11.7%) eventually developed COVID-19, highlighting the importance of identifying, quarantining, and monitoring contacts of COVID-19 patients to limit spread. In Maine, symptom monitoring was not feasible without the use of an automated symptom monitoring tool. Using a tool that permitted enrollees to specify a method of symptom monitoring was well received, because the majority of persons monitored (96.4%) agreed to report using this system.

Public health investigators interviewed persons with COVID-19 upon report of the case to Maine CDC to collect information about their contacts, including date of last exposure. Contacts were defined as persons who were within 6 feet of an infectious person^†^ for ≥15 minutes (≥30 minutes before May 29). Data were stored in the National Electronic Disease Surveillance Base System (NBS)^§^ and sent to Maine CDC’s contact tracing team within 24 hours, along with contact data reported to Maine CDC by other jurisdictions and CDC’s Division of Global Migration and Quarantine. The contact tracing team telephoned contacts to provide quarantine recommendations,^¶^ enroll them in Sara Alert, and instruct them to report symptoms daily via the Sara Alert questionnaire for the remainder of their quarantine. If contacts refused automated monitoring or could not be enrolled because of language barriers, they would be monitored using direct monitoring. Per the Council of State and Territorial Epidemiologists’ case definition,** monitored signs and symptoms included cough, difficulty breathing, fever, chills, shaking with chills (rigors), muscle pain, headache, sore throat, and new loss of taste or smell. The contact tracing team attempted to directly monitor contacts who refused or were unable to be enrolled. Maine CDC staff members conducted case investigations for all enrollees who sought SARS-CoV-2 molecular testing and had a positive result (confirmed cases) irrespective of symptoms and those who did not have molecular testing but reported symptoms (probable cases). Staff members attempted to call or text enrollees who did not respond to the questionnaire within 24 hours. Enrollees who did not report symptoms during their quarantine period were automatically released from quarantine by a Sara Alert–issued notice. Data for contacts enrolled during May 14–June 26, 2020, were extracted from Sara Alert. Enrollee demographic characteristics and Sara Alert program preferences, selected by enrollees at the time of enrollment, were analyzed, and the number of persons enrolled per household were calculated based on self-reported address.

All persons enrolled in Sara Alert during the study period were matched to NBS records using date of birth and the first initial of their first and last names. NBS data were extracted on July 10 to allow contacts enrolled by June 26 to complete 14 days of quarantine. Data extracted from NBS included case status (confirmed or probable), hospitalization status, and outcome, including death. For most analyses, confirmed and probable cases were combined. SAS (version 9.3; SAS Institute) was used to conduct analyses. This activity was determined to meet the requirements of public health surveillance as defined in 45 CFR 46.102(l)(2).

During May 14–June 26, 2020, Maine enrolled 1,622 contacts (enrollees) of 614 COVID-19 patients in Sara Alert. The average number of enrollees per index patient was 2.9 (range = 0–31). Among enrollees, median age was 29 years (range = 0–93 years); 766 (50.3%) were female (Table 1). Race data were available for 1,240 (76.4%) enrollees, 732 (59.0%) of whom identified as white and 486 (39.2%) as black/African American. Ethnicity data were available for 1,020 (62.9%) enrollees, 42 (4.1%) of whom identified as Hispanic/Latino. Primary language was documented for 1,230 (75.8%) enrollees; 985 (80.1%) primarily spoke English, 86 (7.0%) French, and 81 (6.6%) Somali. 

**TABLE 1:** Characteristics of contacts* of confirmed or probable COVID-19^†^ patients enrolled in an automated, web-based symptom monitoring tool (Sara Alert) — Maine, May 14–June 26, 2020

Characteristic	No. (%)
**Total no. of persons enrolled**	**1,622 (100)**
**Age at enrollment, yrs, median (range)**	29 (0–93)
**Sex**	
Female	766 (50.3)
Male	757 (49.7)
Not reported	99 (—)
**Race**	
American Indian/Alaska Native	5 (0.4)
Asian/Pacific Islander	17 (1.4)
Black/African American	486 (39.2)
White	732 (59.0)
Not reported	382 (—)
**Ethnicity**	
Hispanic or Latino	42 (4.1)
Not Hispanic or Latino	978 (95.9)
Not reported	602 (—)
**Primary language**	
American Sign Language	6 (0.5)
Arabic	8 (0.7)
English	985 (80.1)
French	86 (7.0)
Kirundi	8 (0.7)
Lingala	11 (0.9)
Portuguese	10 (0.8)
Somali	81 (6.6)
Spanish	19 (1.5)
Other	16 (1.4)
Not reported	392 (—)
**County**	
Androscoggin	421 (26.9)
Aroostook	39 (2.5)
Cumberland	713 (45.6)
Franklin	12 (0.8)
Hancock	2 (0.3)
Kennebec	60 (3.8)
Knox	1 (0.1)
Lincoln	8 (0.5)
Oxford	32 (2.1)
Penobscot	17 (1.1)
Sagadahoc	25 (1.6)
Somerset	9 (0.6)
Waldo	3 (0.2)
Washington	14 (0.9)
York	193 (12.4)
Out of state	10 (0.6)
Missing	59 (—)

**Abbreviation:** COVID-19 = coronavirus disease 2019.

* Defined as persons who were within 6 feet of an infectious person (symptomatic persons, 2 days before symptom onset to at least 10 days following symptom onset; asymptomatic persons, 2 days before collection of a specimen that resulted in a positive test to 10 days following specimen collection date) for ≥15 minutes (≥30 minutes before May 29).

^†^ Probable cases had either clinical criteria or epidemiologic evidence of exposure (contact with a person with a confirmed or probable COVID-19 case or contact with a person with clinically compatible illness or linkage to a person with confirmed COVID-19), or met vital records criteria (a death certificate listing COVID-19 or SARS-CoV-2 as a cause of death or a significant condition contributing to death with no confirmatory laboratory testing performed for COVID-19). Confirmed cases had confirmatory laboratory evidence of SARS-CoV-2 infection. COVID-19 signs and symptoms included cough, difficulty breathing, fever, chills, shaking with chills (rigors), muscle pain, headache, sore throat, and new loss of taste or smell.

Overall, 475 (29.3%) of 1,622 enrollees were enrolled within 2 days of their last exposure to the patient (Table 2), including 153 (9.5%) enrolled the day of their last exposure, likely indicating ongoing exposure. Among enrollees, 1,564 (96.4%) agreed to be monitored using the automated symptom monitoring, whereas 58 (3.6%) required direct monitoring. Enrollees using automated symptom monitoring preferred text message (976; 60.2%), followed by texted weblink (342; 21.1%), telephone call (127; 7.8%), and e-mailed weblink (119; 7.3%). Most enrollees (870; 59.0%) preferred an evening contact time.

**TABLE 2:** Enrollment details and monitoring preferences among contacts* of confirmed or probable COVID-19^†^ patients enrolled in an automated, web-based symptom monitoring tool (Sara Alert) — Maine, May 14–June 26, 2020

Characteristic	No. (%)
**Total**	**1,622 (100)**
**Interval from last exposure to enrollment (days)**	
0	153 (9.5)
1	47 (2.9)
2	275 (17.1)
3	153 (9.5)
4	208 (12.9)
5	166 (10.3)
6	163 (10.1)
≥7	447 (27.7)
Missing date of last exposure	10 (—)
**Preferred contact method**	
E-mailed weblink	119 (7.3)
Text message	976 (60.2)
Texted weblink	342 (21.1)
Telephone call	127 (7.8)
Direct monitoring^§^	58 (3.6)
**Preferred contact time**	
Morning	479 (32.5)
Afternoon	126 (8.5)
Evening	870 (59.0)
Not recorded	147 (—)
**No. of persons in household enrolled** ^¶^	
1	673 (70.6)
2	125 (13.1)
3	75 (7.9)
4	33 (3.5)
5	21 (2.2)
≥6	27 (2.7)

**Abbreviation:** COVID-19 = coronavirus disease 2019.

* Defined as persons who were within 6 feet of an infectious person (symptomatic persons, 2 days before symptom onset to at least 10 days following symptom onset; asymptomatic persons, 2 days before collection of a specimen that resulted in a positive test to 10 days following specimen collection date) for ≥15 minutes (≥30 minutes before May 29).

^†^ Probable cases had either clinical criteria or epidemiologic evidence of exposure (contact with a person with a confirmed or probable COVID-19 case or contact with a person with clinically compatible illness or linkage to a person with confirmed COVID-19), or met vital records criteria (a death certificate listing COVID-19 or SARS-CoV-2 as a cause of death or a significant condition contributing to death with no confirmatory laboratory testing performed for COVID-19). Confirmed cases had confirmatory laboratory evidence of SARS-CoV-2 infection. COVID-19 signs and symptoms included cough, difficulty breathing, fever, chills, shaking with chills (rigors), muscle pain, headache, sore throat, and new loss of taste or smell.

^§^ Direct monitoring refers to contacts who did not want to be, or could not be, enrolled for automated monitoring. For these contacts, Maine CDC staff members called contacts daily until the end of their quarantine period.

^¶^ Based on address reported at time of enrollment.

Among all enrollees, 231 (14.2%) reported symptoms or had a positive test result. Among these enrollees, 41 (17.7%) were determined not to have COVID-19, including 24 who received negative test results and 17 whose symptoms did not meet those specified by the case definition; these 41 enrollees were reenrolled in Sara Alert for the remainder of their quarantine. Among all enrollees, 190 (11.7%) met the COVID-19 case definition. Among these 190 persons, 127 (66.8%) were confirmed to have COVID-19, and 63 (33.2%) were considered to have probable cases (Table 3). Among all persons with probable and confirmed cases, median age was 32 years (range = 0–93 years); 99 (52.1%) were female. Race data were available for 186 (97.9%) patients, among whom 98 (52.7%) identified as white and 81 (43.5%) as black/African American. Ethnicity was available for 182 (95.8%) patients, six (3.3%) of whom identified as Hispanic/Latino. Exposure was self-reported for 165 (86.8%) patients; household exposure was most common (112; 67.9%). COVID-19 symptoms were reported for 136 (74.3%) patients. Four (2.1%) patients were hospitalized, and one (0.5%) died. During May 14–July 10, Maine reported 1,869 total COVID-19 cases^††^; thus, approximately 10% of Maine’s COVID-19 patients were identified among Sara Alert enrollees.

**TABLE 3:** Characteristics of contacts* of confirmed or probable COVID-19^†^ patients enrolled in an automated, web-based symptom monitoring tool (Sara Alert) who developed COVID-19 during quarantine—Maine, May 14–June 26, 2020

Characteristic	No. (%)^§^
**Total persons with COVID-19**	**190 (100)**
**Case status**	
Confirmed	127 (66.8)
Probable	63 (33.2)
**Reported symptoms**	
Yes	136 (74.3)
No	47 (25.7)
Missing	7 (—)
**Age, yrs, median (range)**	32 (0–93)
**Sex**	
Female	99 (52.1)
Male	91 (47.9)
**Race**	
Asian/Pacific Islander	3 (1.6)
Black/African American	81 (43.5)
White	98 (52.7)
Other	4 (2.2)
Unknown	4 (—)
**Ethnicity**	
Hispanic or Latino	6 (3.3)
Not Hispanic or Latino	176 (96.7)
Missing	8 (—)
**Self-reported exposure settings** ^¶^	
Household	112 (67.9)
Community	29 (17.6)
Health care	26 (15.8)
Unknown	25 (—)
**Hospitalized**	
Yes	4 (2.1)
No	186 (97.9)
**Died from COVID-19**	
Yes	1 (0.5)
No	189 (99.5)

**Abbreviation:** COVID-19 = coronavirus disease 2019.

* Defined as persons who were within 6 feet of an infectious person (symptomatic persons, 2 days before symptom onset to at least 10 days following symptom onset; asymptomatic persons, 2 days before collection of a specimen that resulted in a positive test to 10 days following specimen collection date) for ≥15 minutes (≥30 minutes before May 29).

^†^ Probable cases had either clinical criteria or epidemiologic evidence of exposure (contact with a person with a confirmed or probable COVID-19 case or contact with a person with clinically compatible illness or linkage to a person with confirmed COVID-19), or met vital records criteria (a death certificate listing COVID-19 or SARS-CoV-2 as a cause of death or a significant condition contributing to death with no confirmatory laboratory testing performed for COVID-19). Confirmed cases had confirmatory laboratory evidence of SARS-CoV-2 infection. COVID-19 signs and symptoms included cough, difficulty breathing, fever, chills, shaking with chills (rigors), muscle pain, headache, sore throat, and new loss of taste or smell.

^§^ Percentage calculated among enrollees with nonmissing information.

^¶^ Two contacts reported multiple exposure types.

## Discussion

Contact tracing and symptom monitoring encourages exposed persons to quarantine while providing health departments an opportunity to promptly and proactively identify symptomatic persons, likely reducing SARS-CoV-2 transmission (*5*). Because contact tracing can be resource intensive, using an automated symptom monitoring tool can reduce needed resources (*9*). Contact tracing and the resulting postexposure quarantine and monitoring identified 190 (10%) of Maine’s 1,869 reported COVID-19 cases during May 14–July 10.

These findings suggest that using a symptom monitoring tool with options to accommodate enrollees’ preferences for monitoring method, time of day, and language, might be important for increasing enrollment and improving contact monitoring. Almost all (96.4%) monitored contacts chose automated over direct symptom monitoring. For most of this study period, Sara Alert provided messages in English only, with Spanish added June 10. Enrollees spoke a variety of languages, and French and Somali options were added after this study concluded. 

Although the use of automated symptom monitoring tools might reduce staffing and resources needed to conduct active monitoring of contacts, there continues to be a considerable workload associated with contact enrollment, direct monitoring for nonparticipating contacts and follow-up of nonrespondents (*10*). Maine CDC dedicates approximately 500 person-hours each week to enrolling and monitoring contacts using Sara Alert. Substantial human resources will likely be required to operate any contact tracing and monitoring program. By identifying options that meet communication and accessibility needs of their specific populations, jurisdictions can maximize available resources. However, continued support for jurisdictions to build and maintain contact tracing capacity is needed.

The findings in this report are subject to at least four limitations. First, determining the overall number of contacts identified by all Maine cases was not possible. Contact records in NBS sometimes referenced locations rather than persons, some contacts had no working telephone number or accompanying e-mail address, and an untracked number of contacts refused monitoring, so were not enrolled. Thus, enrollees described in this analysis do not represent the total number of contacts of COVID-19 patients in Maine. Second, during the study period, Sara Alert data extracts did not distinguish between contacts lost to follow-up and those removed based on symptom reporting, making compliance difficult to ascertain. Third, enrollees were not required to be tested for SARS-CoV-2, therefore enrollees with asymptomatic COVID-19 who were not tested were not identified as cases. Finally, although each person was given guidance on quarantine recommendations, adherence was not assessed and is unknown.

Using digital tools in support of a comprehensive contact tracing strategy can make the contact tracing and monitoring process faster and more efficient, as well as provide epidemiologic and clinical data which might result in an improved understanding of COVID-19. Although most contacts in communication with Maine CDC opted to enroll in automated symptom monitoring, the contact tracing program, including contact identification, communication, and monitoring, continues to require resources, including staffing. Automated monitoring tools can augment traditional contact tracing; however, they cannot take the place of a large, trained public health workforce required for a comprehensive COVID-19 response.

**Summary**

**What is already known about this topic?**

Identification and quarantine of contacts of COVID-19 patients can reduce SARS-CoV-2 transmission.

**What is added by this report?**

Maine found that using automated symptom monitoring as a part of the state’s contact tracing program was well received, with the majority of monitored contacts (96.4%) agreeing to automated symptom monitoring. Automated symptom monitoring promptly identified COVID-19 diagnoses among monitored contacts. Among 1,622 persons enrolled into an automated symptom monitoring system, 190 (11.7%) developed COVID-19.

**What are the implications for public health practice?**

Prompt case investigation can rapidly identify contacts and recommend quarantine, reducing additional exposures and transmission. Automated tools, available in multiple languages and formats, might improve contact tracing programs and reduce resource needs, including staffing.

## Footnotes

* https://www.saraalert.org.

† For symptomatic persons, this was defined as 2 days before symptom onset to at least 10 days following symptom onset. For asymptomatic persons, this was defined as 2 days before collection of a specimen that resulted in a positive test to 10 days following specimen collection date.

§ Maine’s National Electronic Disease Surveillance Base System is a local installation and configuration of CDC's National Electronic Disease Surveillance Base System. https://www.cdc.gov/nbs/overview/index.html.

¶ https://www.cdc.gov/coronavirus/2019-ncov/if-you-are-sick/quarantine.html.

** https://wwwn.cdc.gov/nndss/conditions/coronavirus-disease-2019-covid-19/case-definition/2020/.

††
https://www.maine.gov/dhhs/mecdc/infectious-disease/epi/airborne/coronavirus/data.shtml .
